# Diosmin Mitigates Cyclophosphamide Induced Premature Ovarian Insufficiency in Rat Model

**DOI:** 10.3390/ijms22063044

**Published:** 2021-03-17

**Authors:** Noha M. Abogresha, Sally S. Mohammed, Marwa M. Hosny, Hoda Y. Abdallah, Ahmed M. Gadallah, Sahar M. Greish

**Affiliations:** 1 Physiology Department, Faculty of Medicine, Suez Canal University, Ismailia 41522, Egypt; sahar.greish@med.suez.edu.eg; 2 Histology and Cell Biology Department, Faculty of Medicine, Suez Canal University, Ismailia 41522, Egypt; sallysalem@med.suez.edu.eg; 3 Medical Biochemistry and Molecular Biology Department, Faculty of Medicine, Suez Canal University, Ismailia 41522, Egypt; marwahosny@med.suez.edu.eg; 4 Oncology Diagnostic Unit lab, Faculty of Medicine, Suez Canal University, Ismailia 41522, Egypt; 5 Department of Histology and Cell Biology (Genetics Unit), Faculty of Medicine, Suez Canal University, Ismailia 41522, Egypt; hoda_ibrahim1@med.suez.edu.eg; 6 Center of Excellence in Molecular and Cellular Medicine, Faculty of Medicine, Suez Canal University, Ismailia 41522, Egypt; 7 Obstetrics and Gynecology Department, Faculty of Medicine, Suez Canal University, Ismailia 41522, Egypt; Ahmed_gadallah@med.suez.edu.eg; 8 Physiology Department, School of Medicine, Badr University in Egypt (BUC), Badr 11829, Egypt

**Keywords:** premature ovarian insufficiency, miRNA-145, oxidative stress

## Abstract

The current study was designed to investigate the protective role of diosmin against cyclophosphamide-induced premature ovarian insufficiency (POI). Female Swiss albino rats received a single intraperitoneal dose of cyclophosphamide (200 mg/kg) followed by 8 mg/kg/day for the next 15 consecutive days either alone or in combination with oral diosmin at 50 or 100 mg/kg. Histopathological examination of ovarian tissues, hormonal assays for follicle stimulating hormone (FSH), estradiol (E2), and anti-Mullerian hormone (AMH), assessment of the oxidative stress status, as well as measurement of the relative expression of miRNA-145 and its target genes [vascular endothelial growth factor B *(VEGF-B)* and regulator of cell cycle (*RGC32*)] were performed. Diosmin treatment ameliorated the levels of E2, AMH, and oxidative stress markers. Additionally, both low and high diosmin doses significantly reduced the histopathological alterations and nearly preserved the normal ovarian reserve. MiRNA-145 expression was upregulated after treatment with diosmin high dose. miRNA-145 target genes were over-expressed after both low and high diosmin administration. Based on our findings, diosmin has a dose-dependent protective effect against cyclophosphamide-induced ovarian toxicity in rats.

## 1. Introduction

Ovarian folliculogenesis (comprising primordial follicle assembly, follicle growth, ovulation, and formation of the corpus luteum) is a dynamic and highly regulated process that determines female reproduction [1]. Abnormalities in folliculogenesis result in serious ovarian pathological conditions such as premature ovarian insufficiency (POI) and polycystic ovary syndrome [2,3].

Most follicles undergo degeneration during growth and development in a process known as follicular atresia. Abnormal follicular atresia may accelerate follicular depletion and lead to ovarian insufficiency. Recent studies have suggested that granulosa cell (GC) apoptosis is the main cause of follicular atresia [4,5,6]. Oxidative stress, which arises as a consequence of the excessive production of reactive oxygen species (ROS) and/or impaired antioxidant defense mechanisms, is believed to be the major causes of GC apoptosis [7].

Cyclophosphamide is a chemotherapeutic agent widely used in the treatment of variable types of cancer [8]. It is a highly effective agent, used alone or in combination with other chemotherapeutics, despite its hepatic [9], renal [10], nervous [11], and cytotoxic side effects [12]. With the current increasing incidence of cancer among young women, chemotherapy-induced gonadotoxicity becomes a persisting cause of POI. Cyclophosphamide has a deleterious effect on female reproductive organs, especially the ovary [13]. Toxic effects of cyclophosphamide are related to its active metabolites which bind to DNA, disrupting DNA synthesis, and produce reactive oxygen species (ROS) by conjugation with glutathione, interfering with the antioxidant defense system of the ovary. Moreover, malondialdehyde (MDA), a marker of lipid peroxidation, is increased following cyclophosphamide treatment [13].

Diosmin is a natural flavone glycoside found mainly in citrus [14]. It shows anti-hyperglycemic [15], anti-cancer [16], anti-inflammatory, and anti-oxidant-like actions [17]. These effects are achieved via decreasing the levels of hydroxyl free radicals, increasing the free thiol (SH-) group concentration and the natural scavenger capacity [18,19,20,21,22].

MicroRNAs (miRNAs) are small (20–24 nucleotides) non-coding endogenous RNAs that play a fundamental role in multiple biological processes and pathology through binding to multiple messenger RNA (mRNA) targets [23]. MiRNAs thus modulate transcription or post-transcription expression of their target genes. MiRNAs are involved in the entire process of ovarian follicle development, including follicle growth, atresia, and ovulation [3,23,24]. It has been shown that inactivation of Dicer1 (an RNA-specific endoribonuclease (involved in the regulation of miRNA) in follicular GCs leads to an increase in degenerated follicles, suggesting an essential role for miRNAs in the regulation of GC function [24].

miRNA-145 is one of the most predominant miRNA populations in the ovary [3]. MiRNA-145 has been reported to protect cardiomyocytes against oxidative stress-induced apoptosis by targeting the mitochondrial apoptotic pathway [25]. In addition, miRNA-145 expression is associated with the response of vascular smooth muscle cells to hydrogen peroxide (H_2_O_2_)-induced oxidative stress [26]. Together, these data indicate that miRNA-145 may participate in the regulation of the oxidative stress-induced apoptotic pathway. Bioinformatics and gene ontology analysis revealed that the target genes of the predominantly expressed ovarian miRNAs are involved in cell cycle regulation, cellular growth, proliferation, apotosis, and other ovarian functions [27].

No previous studies, to the best of our knowledge, have investigated the protective effect of diosmin against ovarian cycphosphamide toxicity. Therefore, in this study, we developed a cyclophasamide-induced POI model in female Swiss albino to investigate the potential protective role of diosmin in cyclophosphamide-induced POI, through assessing histopathological changes in ovarian tissues, the oxidative stress status, hormonal assays for follicle-stimulating hormone (FSH), estradiol (E2), and anti-Mullerian hormone (AMH) and the expression levels of miRNA-145 and its target genes [vascular endothelial growth factor B *(VEGF-B)* and regulator of cell cycle (*RGC32*)].

## 2. Results

### 2.1. Effect of Cyclophosphamide on Ovarian Morphology

All rats were weighed before and after cyclophosphamide treatment. No significant difference was observed. However, a significant reduction in the size of the ovaries in the POI (G II) group after cyclophosphamide administration was noted (Table 1).

### 2.2. Chemotherapy-Induced Serum Hormonal Level Changes in the POI Model

Serum levels of FSH, E2, and AMH were significantly different among the study groups (GI, G II, G III, G IV) (*p* = 0.001, *p* = 0.032, *p* < 0.0001, and *p* < 0.0001, respectively). Serum FSH level in the POI (G II) group was increased compared to that in the control (G I), diosmin 50 (G III), and diosmin 100 (G IV) groups (*p* = 0.001, *p* < 0.0001, and *p* = 0.016, respectively). Serum E2 and AMH levels in the POI (G II) group were markedly decreased compared to that in the control (G I) (𝑝 < 0.0001 and *p* = 0.016, respectively), diosmin 50 (G III), (*p* = 0.019 and *p* < 0.0001, respectively), and diosmin 100 (G IV) groups (*p* = 0.012 and *p* < 0.0001, respectively) (Table 2).

### 2.3. Oxidative Stress Markers in the POI Model

Serum MDA level and catalase activity were measured in the study groups. Serum MDA level was markedly higher in POI (G II) rats as compared to the control (G I) and diosmin-treated (GIII and G IV) groups. Catalase activity, on the other hand, was not significantly different among the study groups (Table 3).

### 2.4. Effect of Cyclophosphamide and Diosmin on Follicular Growth

#### 2.4.1. Hematoxylin and Eosin staining results

##### The Control Group (Group I, Figure 1, Figure 2, Figure 3 and Figure 4)

Microscopic examination of the ovaries from the control (G I) group revealed normal ovarian structure. Multiple follicles in different stages of development were observed (Table 4). These included several primordial follicles with central oocytes and nuclei, primary follicles with larger oocytes, and secondary follicles with a big oocyte surrounded by a layer of cubical cells overlying a zona pellucida (ZP), multiple layers of GCs and a cavity. All follicles were surrounded by flat stromal cells. The sections also showed multiple mature Graafian follicles (GF) with normal wall layers. The cumulus oophorus (CO) comprised one big oocyte with a prominent nucleus, surrounded by a ZP, Corona radiata (CR) and GCs. The corpus luteum (CL) exhibited large faintly stained acidophilic cells.

##### POI Group (Group II, Figure 5)

Ovarian sections of POI (G II) group rats showed a significant decrease in the total follicle count (Table 4) with a significant reduction in the number of primordial and primary follicles (0.5 ± 0.52), as well as antral and mature follicles (0.4 ± 0.41, 0.2 ± 0.22, respectively) in comparison with the control (G I) and diosmin-treated (G III and G IV) groups. Graafian follicles exhibited edema and separation of cells, loss of demarcation between the layers forming their walls and loss of the CO. The GCs were shrunken with pyknotic nuclei. There was a remarkable increase in the number of atretic follicles compared to other groups (2.8 ± 0.63). The ovarian medulla was also destructed with massive edema.

##### Diosmin 50 (Group III, Figure 6)

Sections from the diosmin 50 (G III) group revealed some improvement compared to the POI (G II) group. There was an increase in the total number of follicles (Table 4). The ovarian cortex had different stages of follicular development with little stroma in between. There was a significant increase in the number of follicles: primordial (2 ± 0.86), primary (2.5 ± 0.84), antral (1.5 ± 0.7), and GF (0.7 ± 0.48) compared to the POI (G II) group. There was also a significant decrease in the number of atretic follicles (1 ± 0.8). The corpus luteum was also normal with faint acidophilic cells.

##### Diosmin 100 (Group IV, Figure 7)

Ovarian sections from the diosmin 100 (G IV) group revealed normal architecture comparable to those from the control (G I) group. There was an increase in the total number of follicles (Table 4) compared to G II and G III. The ovarian cortices had many normal follicles in different stages of development.

#### 2.4.2. Masson’s Trichrome Staining Results

##### The control (G I) Group

**Figure 8:**
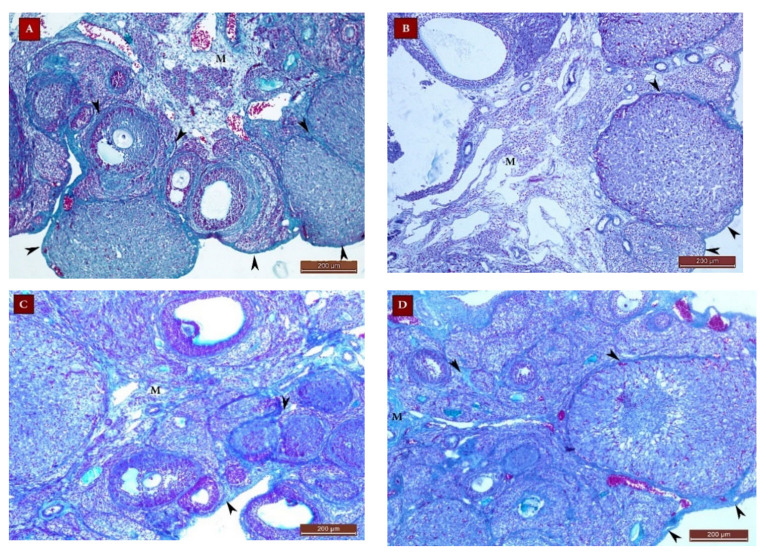
Masson’s trichrome stain ×100 photomicrographs of ovaries from all study groups. (**A**) Ovarian section from the control (G I) group showing normal collagen (green color) distribution in the tunica albuginea and in the cortex between and surrounding the growing follicles (arrow heads) and in the medulla (M). (**B**) Section from the POI (G II) group showing a decrease in the collagen content at the ovarian surface and in the cortex and M. Sections from the diosmin 50 (G III) group (**C**) and diosmin 100 (G IV) group (**D**) showing restoration of the collagen content at the ovarian surface and in the cortex and medulla.

Ovarian sections from the control (G I) group stained with Masson’s trichrome revealed normal collagen content and distribution at the ovarian surface (tunica albuginea) and in between and surrounding the growing follicles in the cortex and in the medulla (Figure 8A). The mean color area percentage of collagen was 29.49 ± 4.7.

##### The POI (G II) Group

**Figure 9:**
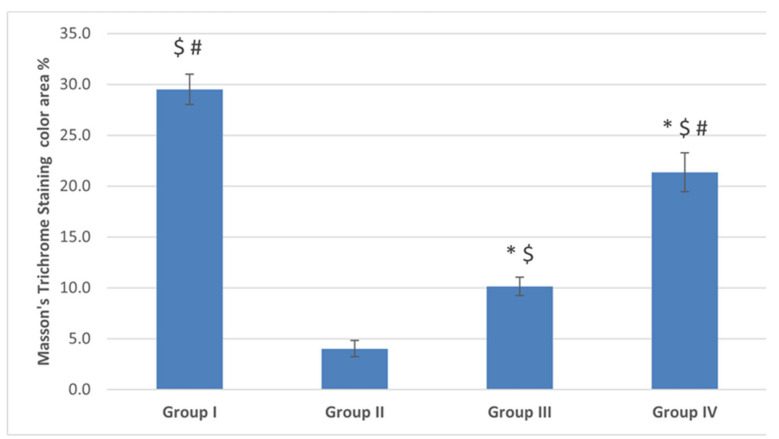
The mean color area percentage of the green stained collagen. Data are expressed as means ± SEM (*n*= 6) and analyzed using one-way ANOVA followed by post-hoc Bonferroni multiple comparison test. (Symbols: *: *p* ≤ 0.05 compared to the control group (Group I); $: *p* ≤ 0.05 compared to the POI group (Group II); #: *p* ≤ 0.05 compared to diosmin 50 group (Group III)).

Ovarian sections from this group exhibited a significant decrease in the collagen content compared to group I, all over the ovarian sections (Figure 8B). The mean color area percentage of collagen was 4.03 ± 2.5 (Figure 9).

##### Diosmin 50 (Group III) and Diosmin 100 (Group IV) Groups

Sections from the diosmin-treated groups (G III and G IV) revealed restoration of the collagen content all over the ovarian sections, compared to the POI (G II) group (Figure 8C,D). There was a significant increase in the mean color area percentage of collagen compared to the POI (G II) group (Figure 9). The mean color area percentage of collagen in group III was 10.13 ± 2.8 (Figure 9). Moreover, the collagen content in group IV was increased compared to group III and amounted to 21.36 ± 5.94 (Figure 8C,D, Figure 9).

#### 2.4.3. Immunostaining Results

##### The Anti-Caspase 3

**Figure 10:**
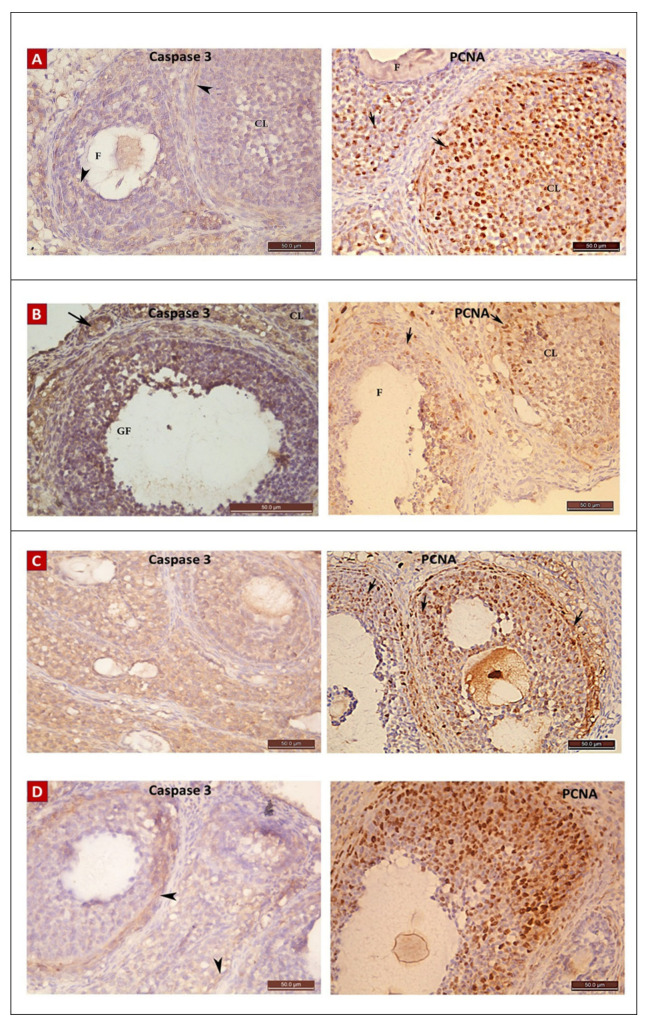
Anti-caspase 3 and anti-PCNA immunostaining ×400 photomicrographs in ovaries of all study groups. Immunostained ovarian sections from control (G I) (**A**), POI (G II) (**B**), diosmin 50 (G III) (**C**), and diosmin 100 (G IV) (**D**) groups. Anti-caspase 3 immunostaining appears as deep brown cytoplasmic reaction in B ovary, while little reaction is apparent in A, C, and D. The anti-PCNA reaction showed many positive brown nuclear reactions in A, C, and D with lesser number in B.

**Figure 11:**
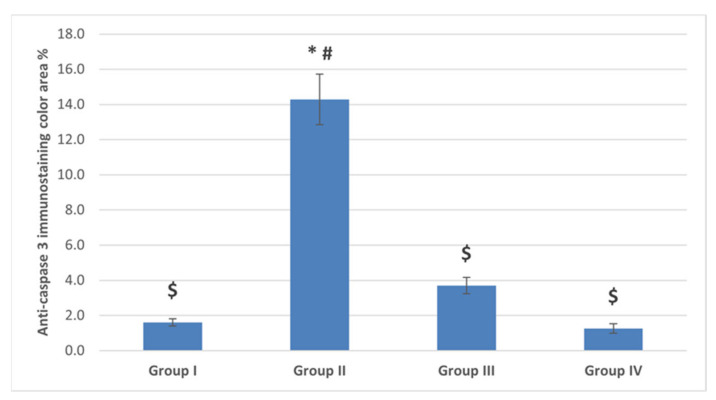
The mean color area percentage of anti-caspase 3 immunostaining. Data are expressed as means ± SEM (*n*= 6) and analyzed using one-way ANOVA followed by post-hoc Bonferroni multiple comparison test. (Symbols: *: *p* ≤ 0.05 compared to the control group (Group I); $: *p* ≤ 0.05 compared to the POI group (Group II); #: *p* ≤ 0.05 compared to diosmin 50 group (Group III)).

Ovarian sections from the POI (G II) group immune-stained for caspase 3 exhibited brown cytoplasmic reaction in cells of the granulosa, TI and stromal cells (Figure 10) with a significant increase in the mean color area percentage (14.3 ± 6.4) compared to the control (G I) and diosmin-treated (G III and G IV) groups (Figure 11).

##### The Anti-PCNA

**Figure 12:**
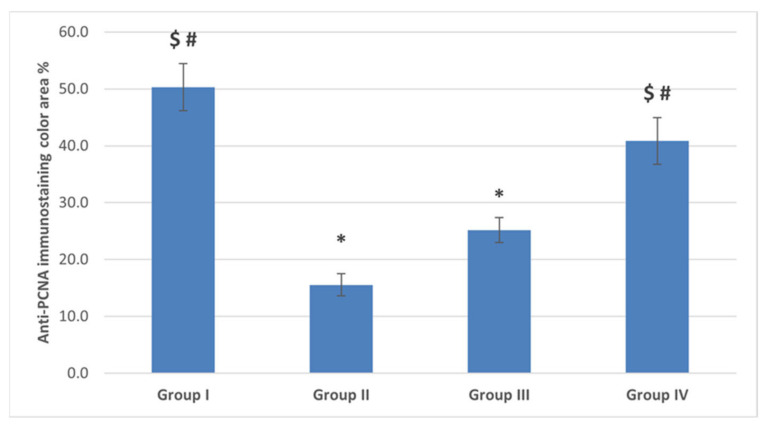
The mean color area percentage of PCNA immunostaining. Data are expressed as means ± SEM (*n*= 6) and analyzed using one-way ANOVA followed by post-hoc Bonferroni multiple comparison test. (Symbols: *: *p* ≤ 0.05 compared to the control group (Group I); $: *p* ≤ 0.05 compared to the POI group (Group II); #: *p* ≤ 0.05 compared to diosmin 50 group (Group III)).

Ovarian sections from the POI (G II) group had a remarkably lower mean color area percentage (15.6 ± 8.74) compared to the control (G I) group. Moreover, the color area percentage of the proliferating cells was significantly increased with diosmin high dose in the diosmin 100 (G IV) group (40.85 ± 18.29) compared to the POI (G II) and diosmin 50 low dose (G III) group (Figure 10 and Figure 12).

### 2.5. Cyclophosphamide or Diosmin Effect on MiRNA-145 and Target Genes

**Figure 13:**
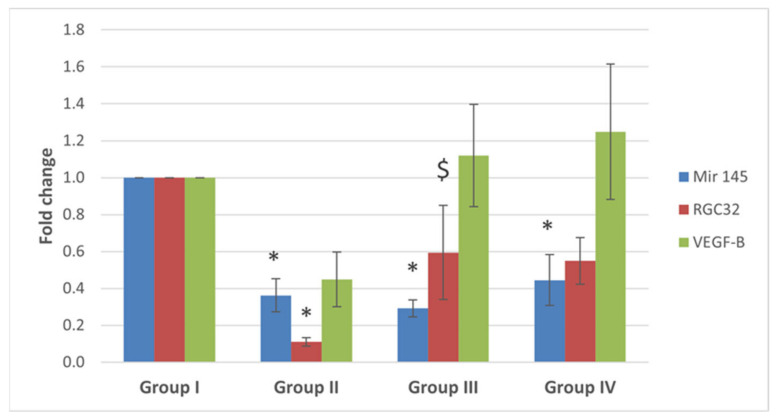
Fold change for miRNA-145, *RGC32* and *VEGF-B* genes expression from real time-poymerase chain reaction results. Data are expressed as means ± SEM (*n*= 6) and analyzed using one-way ANOVA followed by post-hoc Bonferroni multiple comparison test. (Symbols: *: *p* ≤ 0.05 compared to the control group (Group I), and $ indicates *p* ≤ 0.05 compared to POI group (Group II)).

MiRNA-145 was markedly under-expressed in the ovaries from the POI (G II) group and both of low and high diosmin-treated (G II and G IV) groups compared to the control (G I) group (*p* < 0.0001). Gene expression profiling for the miRNA-145 target genes showed significant under-expression of *RGC32* in the POI (G II) group compared to the control (G I) group (*p* = 0.006), and over-expression in the low diosmin-treated (G III) group compared to that of the POI (G II) group (*p* = 0.038). On the other hand, *VEGF-B* expression levels were higher, though non-significant, in both low and high diosmin-treated (G III and G IV) groups compared to the control (G I) and POI (G II) groups (*p* = 0.118), as shown in (Figure 13).

## 3. Discussion

A literature survey retrieved no scientific reports on the protective effects of diosmin against cyclophosphamide ovarian toxicity. This study highlights the protective role of diosmin against cyclophosphamide induced ovarian tissue injury. In experimental rats, diosmin was able to significantly reduce the biochemical and histological alterations, induced by cyclophosphamide or other chemicals in tissues other than ovary [28,29,30,31]. In cancer female patients, fertility preservation is a major concern taking into consideration that cyclophosphamide is one of the widely used anti-cancer drugs [32]. Rats were dosed with cyclophosphamide (200 mg/kg i.p.) to quantify follicular depletion and evaluate apoptosis. The selected dose depends on Song et al. who observed a significant depletion of the follicular reserve using this dose [33].

Cyclophosphamide causes cell damage, inappropriate cell division, and apoptosis of the cells [34,35], in the same context, we found that cyclophosphamide interferes with the ovarian oxidation system through the production of reactive oxygen species that results in an imbalance in the antioxidant defense system of the ovary and eventually cell apoptosis. The ovarian cells are highly dividable, making them a target for this toxic effect of cyclophosphamide.

In the present study, animals treated with cyclophosphamide had a significant decrease in the total ovarian oocytes count and a significant increase in the number of atretic follicles. There was a significant decrease in the ovarian oocyte reserve from primordial and primary follicles with a significant damage to the mature ones. These findings were concurrent with some studies [34,35,36]. In the same vein, Song et al., 2016 reported that after 4 weeks of administration of cyclophosphamide to rats, there was a significant decrease only in secondary follicles count compared to the control group, and they referred this to the main influencing role of granulosa cells in folliculogenesis which was lost by the toxic effect of the drug [33]. We observed that most of the damage affected folliculogenesis and led to ovarian failure which may be attributed to altered hormonal secretion of the ovarian granulosa cells.

Moreover, we reported a significant depletion of the follicular reserve with a mean follicular loss of 45% at the dose of 200 mg/kg. Our results also revealed affection on the blood vessels with leakage, edema, and destruction of ovarian stroma cells and medulla. Liu et al. (2016) reported similar results [37]. This toxic effect is responsible for the decrease in collagen content in the ovarian stroma, as shown from our findings. Xiong et al.(2016) highlighted that cyclophosphamide significantly increases the expression of P53, P16, and caspase 3 in the granulosa cells of the oocytes, so increases apoptosis in these cells and thus affects folliculogenesis [34,38]. These were in accordance with our findings that had significantly increased the anti-caspase immunostaining in the ovarian granulosa, theca interna and stromal cells and significantly decreased the proliferation activity of the cells indicated by the anti-proliferation cell nuclear antigen immunostaining, which revealed significant decrease in the number of proliferating cells compared to the control group.

Diosmin effects is dose dependent [39,40], so we used two different doses of diosmin (50 and 100 mg/kg) based on its anti-inflammatory and anti-oxidant evidence [39,40]. Diosmin succeeded to display a significant increase in the total count of oocytes with different stages of folliculogenesis in the ovarian cortices, compared to the cyclophosphamide treated group, and a decrease in the atretic follicles count. These findings were dose dependent. Additionally, there were normal ovarian stroma and medulla. Around 20% of healthy growing follicles were still observed with diosmin treatment and serum FSH levels were reduced either by low or high dose of diosmin.

Furthermore, the depletion of primordial follicles was not observed after cyclophosphamide diosmin treatment which supports our study hypothesis that diosmin could inhibit follicular growth and FSH secretion to protect ovarian reserve against cyclophosphamide. Until now, there are no published studies similar or in contrary to our results concerning the role of diosmin. The use of diosmin in the present work showed a significant decrease in the immune-reactivity of caspase-3 against cyclophosphamide, with a significant increase in the immune-reactivity of the proliferation nuclear antigen of the ovarian oocyte cells. Similar findings of the anti-apoptotic activity of diosmin in acetic acid-induced ulcerative colitis were reported [41]. Our findings are mostly mediated via diosmin antioxidant and anti-apoptotic effects as shown by the decrease in oxidative stress markers (MDA), enhanced antioxidant defense mechanism as catalase (CAT), the reduction in the necrosis and vacuolar degeneration on histopathological examination after diosmin treatment.

In our study, the POI group showed a typical reduction in follicle pool, diminished ovarian reserve and subsequent follicular dysfunction expressed as an increased FSH level. GCs mainly produce E2, over-apoptosis induced by cyclophosphamide as shown results in a decreased E2 level. Normally, E2 exerts negative feedback on FSH production in the hypothalamic pituitary axis [42]. We reported a decreased E2 level which leads to a non-significant increase in the FSH level. In the same vein, AMH reduced levels can be explained on the following basis: Firstly, AMH is expressed by GCs of the primary follicles or early antral follicles [43]. Secondly, the level of AMH correlates with the size of the follicle pool [44] which showed 45% reduction in our study. These hormonal levels especially AMH reflect the depleted follicular storage in POI group. On the other hand, AMH can be a reliable predictor of POI improvement in diosmin treatment groups other than FSH and E2 [45,46]. The preservation of the levels of FSH, E2, and AMH near normal by diosmin high and low doses came in accordance with our histological and immunohistochemical findings improvement.

Dysregulation of microRNAs is proved to alter the rate of granulosa cell (GC) proliferation contributing to abnormal folliculogenesis. Post-transcriptional inactivation of their target mRNAs through binding by base-pairing and thus inducing either translational repression or target mRNA destabilization. MiRNA-145 is down-regulated in human GCs from PCOS [47]. MiRNA-145 acts as a tumor suppressor and down-regulated in various cancers [48,49,50]. Moreover, miRNA-145 is a novel and promising molecular target for improving the dysfunction of GCs [47] It is considered as one of the most predominant miRNA populations in the ovary [16]. So, we postulated that miRNA-145 dysregulation represents a mechanism by which diosmin can protect the ovarian reserve. Our reported significant down-regulation in miRNA-145 in all study groups in comparison to the control group that ascertains the negative effect of cyclophosphamide on folliculogenesis and subsequently, the ovarian reserve. Only the diosmin high dose miRNA-145 level was up-regulated when compared to the POI group. These findings can obviously explain the histological and functional improvement in our study. Cyclophosphamides act through DNA damage of cancer cells, to some extent, we can defend diosmin possible accusation that it may weaken cyclophosphamide action on tumor cells. Diosmin displays anti-carcinogenic activity in many types of cancer [51] and shows pro-apoptotic activity and chemo-preventive potential in a cancer cell-specific manner [52] through multiple pathways. miRNA-145 acts as a tumor suppressor and down-regulated in various cancers as pointed earlier [48,49,50]. miR-145 inhibits tumor cell proliferation [53], increases tumor cell sensitivity to chemotherapeutic drugs [54].

miRNA-145 up-regulation can suppress cell proliferation by targeting and inhibiting IRS1 (insulin receptor substrate) expression to inhibit PI3K/Akt and MAPK/ERK signaling pathways [55]. Bioinformatics and gene ontology analysis revealed that the target genes of these predominantly expressed miRNAs in the ovary are involved in cell cycle regulation; cellular growth, proliferation, and apoptosis; endocrine system disorders; and ovarian functions [56]. This can be explained by the evidence that diosmin by itself could increase the levels of VEGF-B directly [57] or via another mechanisms.

In conclusion, diosmin used in a dose-dependent manner mitigated cyclophosphamide-induced POI. Diosmin significantly reduced the biochemical as well as the histological alterations induced by cyclophosphamide, because of its antioxidant and anti-apoptotic effects as well as regulation of miRNA-145 and/or overexpression of its target genes. Therefore, diosmin can be a promising agent in the protection against cyclophosphamide-induced ovarian toxicity, especially in the young female patients. Future studies should investigate other mechanisms by which diosmin can alleviate chemotherapeutic agents’ side effects. More studies to address the possibility that diosmin acting systemically, apart from an ovary, may weaken the anti-tumor chemotherapeutic effects.

## 4. Materials and Methods

### 4.1. Experimental Animals

Eight-week-old female Swiss albino rats (weight 150 ± 20 g) were purchased from Moustafa Rashed Company and maintained in polyethylene cages in a clean animal room. Experimental conditions: temperature 20 ± 2 °C; normal light-dark cycle; and continuously available food and water. Animals were acclimatized a week before starting the experiment. All procedures were performed at the Physiology Department, Medical Biochemistry and Molecular Biology Department, and the Oncology Diagnostic Unit, Faculty of Medicine, Suez Canal University (Ismailia, Egypt). This study was performed in accordance with the guide for the care and use of laboratory animals, NIH, Bethesda. Our study protocol was approved by the Institutional Research Ethics Committee of the Suez Canal University, Faculty of Medicine (ID. 4475).

### 4.2. Chemicals

Cyclophosphamide was purchased from Shanxi PUDE pharmaceutical company (Shanxi, China). Diosmin was purchased from Sigma (St. Louis, MO, USA). All the oxidative stress assay kits were purchased from Biodiagnostics Co. (Cairo, Egypt).

### 4.3. Experimental Model of Premature Ovarian Insufficiency

A single dose of cyclophosphamide (200 mg/kg, mimicking the acute exposure to an acute large dose in humans) was injected intraperitoneally, then followed by 8 mg/kg/day for the next 15 consecutive days [33]. Before the beginning of the experiment, a pilot of four rats were scarified to confirm the establishment of the chemotherapy-induced POI rat model. The ovarian tissues showed atrophic changes 24 h after induction.

### 4.4. Study Design

Twenty-four rats were allocated into four groups (6 rats/group): (i) Control group: rats received saline only during the experimental period; (ii) POI group: injected with cyclophosphamide only for POI induction; (iii) Diosmin low dose treated rats (50 mg/kg/BW) for 15 days with concomitant induction of POI using cyclophosphamide [29]. (iv) Diosmin high dose treated rats (100 mg/kg/BW) for 15 days with concomitant induction of POI using cyclophosphamide [53]. After 15 days, rats were anaesthetized and sacrificed; blood samples were collected by direct cardiac puncture for lab parameters. Ovarian tissue was collected for histopathological, immunohistochemistry, and RT-PCR.

### 4.5. Histopathological Examination

The ovaries were fixed in 10% formaldehyde for 24 h, then the specimens were processed in graduated ethyl alcohol to paraffin blocks. The blocks were sectioned at 4–5 µm thickness for the histological and immunohistochemical techniques.

Ovarian sections were stained with hematoxylin and eosin (H&E) (Merck, Rahway, NJ, USA) and examined at 200× magnification using a power light microscope (Zeiss, Germany) to study the general architecture, histopathological changes, and the number of normal growing and atretic follicles of 15 sections per ovary (in randomly chosen fields) using a quantitative analysis. The primordial follicles had small central oocytes surrounded by a single layer of flat granulosa cells (GCs); the primary follicles had a larger oocyte, surrounded by single layer of GCs, and outer flat stromal cells. The secondary or antral follicles had an oocyte covered by zona pellucida (ZP) and surrounded by corona radiata (CR) cells and GCs, with one or more small fluid cavities. The Graafian follicles (GF) were the largest, close to the ovarian surface, comprising a cumulus oophorus (CO), a large single fluid cavity and multiple layers of GCs lining their walls. The CO was shifted to one side, had a large oocyte covered by ZP, CR, and multiple layers of GCs. The GF was also enveloped with the theca interna (TI) and theca externa (TE) layers. The TI cells were deeply stained, while cells of the TE layer were lightly stained. Both layers were separated from the follicle by a basement membrane. The atretic follicles had no oocyte, and exhibited pyknotic/separated GCs, detached CR or ZP.

Ovarian sections were also examined using Masson’s trichrome stain to study the connective tissue content collagen fibers distribution of the ovaries. The amount of collagen was estimated through assessing the color area percentage of the green stained collagen using the Fiji image-J program (version 1.52i, National Institutes of Health, Bethesda, MD, USA).

### 4.6. Immunohistochemistry Staining

Formalin-fixed paraffin-embedded blocks of ovarian tissue were sectioned (4-μm thick) and mounted on positively charged slides, then processed for manual immunohistochemical staining. Sections were incubated with 3% H_2_O_2_ for 10 min, washed with PBS for 5 min, and then incubated with primary antibodies for caspase 3 (Gene Tex, Irvine, CA, USA) to detect the degree of apoptosis and for proliferating cells PCNA (proliferating cell nuclear antigen, ABclonal Technology, Woburn, MA, USA) at 4 °C overnight. Both antibodies were polyclonal rabbit anti-mouse antibodies at dilution of 1:200 for caspase3 and 1:100 for PCNA. Next, the sections were washed with PBS and incubated for 30 min with biotinylated secondary antibodies at room temperature. The staining was completed using a power-stain TM 1.0 poly horseradish peroxidase DAB kit (Genemed Biotechnologies, Torrance, CA USA), and the sections were counter-stained with hematoxylin. The color area percentage of the brown stained cytoplasm in the anti-caspase 3 stain; and the number of the brown stained nuclei in the anti-PCNA stain were examined in 15 random sections per ovary and calculated using the Fiji image-J program, National Institutes of Health, USA.

### 4.7. Serum Biochemical Analysis

The obtained blood samples were left to clot for 30 min, then centrifuged at 3000 rpm for 15 min to obtain clear sera. The sera were then stored at −20 °C for further biochemical analysis.

#### 4.7.1. Oxidative Stress Markers

Oxidative stress markers in the serum were evaluated using a UV/VIS spectrophotometer (Unico S2100 Series, Wixom, MI, USA). Lipid peroxide (malondialdehyde, MDA) assessment: the method was based on the reaction between MDA and thiobarbituric acid (TBA) in acidic pH at a temperature of 95 °C for 30 min to produce TBA reactive substances, then the absorbance was measured at wavelength 534 nm as described by Yagi et al. [58]. Catalase was measured according to the method described by Aebi et al. [59] for measuring decomposed hydrogen peroxide (H_2_O_2_).

#### 4.7.2. Hormonal Assays for FSH, E2, and AMH

FSH, E2, and AMH levels were assessed in serum samples using chemiluminescent detection technology with magnetic particle separation (Beckman Coulter-Access 2 immunoassay system, Irving, TX, USA). A paramagnetic particle, chemiluminescent, two-step enzyme immunoassay for the quantitative determination of hormones in serum using the access immunoassay system. A second separation and wash step remove unbound conjugate. A chemiluminescent substrate, Lumi-Phos 530, is added to the reaction vessel and light generated by the reaction is measured with a luminometer. The photon production is proportional to the amount of the hormones in the sample. The amount of analyte in the sample is determined by means of a stored, multi-point calibration curve.

### 4.8. RNA Isolation, Reverse Transcription, and Target Gene Expression

Total RNA was extracted from a whole fresh ovary that was homogenized with tissue homogenizer in the presence of 700 μL qiazol reagent in accordance with the manufacturer’s protocol (miRNeasy mini-Kit, Cat no. 217004, Qiagen, Hilden, Germany). RNA conversion to complementary DNA was carried out using miScript II RT Kit (Qiagen, Cat no. 218161) and the thermocycler (Robocycler Gradient 96, BIOMETRA^®^, Princeton, NJ, USA). Reactions were run with the following steps using Maxima SYBR Green qPCR master mix (Thermofisher Scientific, USA, cat. No. K0251). The 25 μL PCR included 7.5 μL RT product (diluted to reach a concentration of 20 ng cDNA), 1 μL RNase-free water, 12.5 μL SYBR green, 2 μL of diluted 1:9 (concentration 10 pg) forward primer (willowfort, UK), 2 μL of diluted 1:9 (concentration 10 pg) reverse primer (willowfort, UK), for *RGC32* gene; Forward primer sequence 5′-ACTCCTCGGAAAGCCAAATTA and Reverse 5′-GAATCTCAAACTCCTTGCTTCAC-3′, and for *VEGF-B* gene; Forward primer sequence 5′-GCACAAATCAGATGGTGAGAGA-3′ and Reverse 5′-CAGGAGATGGTTGATGGCTTAG-3′; using Step One™ real-time PCR system (Applied biosystems, Lincoln, USA). The reactions were incubated in a 48-well optical plate at 95 °C for 15 min for initial activation step, followed by 40 cycles of denaturation at 94 °C for 15 s, annealing at 55 °C for 30 s, and extension at 70 °C for 30 s. The fluorescence signal was acquired at 70 °C and compared to the endogenous control *β-actin* (willowfort, UK) Forward primer sequence 5′-TGTGACGTTGACATCCGTAAAG-3′ and Reverse 5′-GGCAGTAATCTCCTTCTGCATC-3′. The Real-Time PCR reactions were performed in accordance with the Minimum Information for Publication of Quantitative Real-Time PCR Experiments (MIQE) guidelines [59]. Fold change of each gene was estimated using LIVAK method (=2^−ΔΔCt^).

### 4.9. microRNA-145 Expression Analysis

The expression level of miRNA-145 was assessed using SYBR Green qPCR analysis and normalized with SNORD68 in ovarian samples. RNA conversion to complementary DNA was carried out using miScript II RT Kit (Qiagen, Cat no. 218161) using thermocycler (Robocycler Gradient 96, BIOMETRA^®^, Princeton, NJ, USA). The following miScript primer assays (Qiagen, Cat no. 218300) were used for miRNA-145 (Thermofisher Scientific, Waltham, Ma, USA, MS00000434), and for SNORD68 (Thermofisher Scientific, USA, MS00033712). In brief, 12.5 μL of miScript SYBR Green PCR master mix (Qiagen, Cat no. 218073), 2.5 μL miScript Primer Assays, 7.5 μL PCR grade water, and 2.5 μL cDNA template diluted to reach concentration of 20 ng cDNA were included in the reaction. The real-time PCR reactions were performed in accordance with the Minimum Information for Publication of Quantitative Real-Time PCR Experiments (MIQE) guidelines [60]. Fold change of miRNA-145 was estimated using LIVAK method (=2^−ΔΔCt^).

### 4.10. In Silico Data Analysis

The miRWalk database was used to predict the target genes for miRNA-145 [61]. Gene and protein data were retrieved from the National Center for Biotechnology Information ([62] and the uniprot databases [63]). The STRING database (Search Tool for the Retrieval of Interacting Genes/Proteins) version 11.0 was used to reveal known and predicted protein-protein interactions between the selected target genes with others, including direct (physical) and indirect (functional) associations [64]. Moreover, STRING was used to analyze the gene ontology terms of biological process, molecular function, and cellular compartment for the selected gene targets. The target genes specific primer assays have been designed and evaluated by us using online Bioinformatics tools and in silico [65].

### 4.11. Statistical Analysis

Statistical tests were performed using the SPSS IBM version 25. Data are expressed as means ± SEM. Statistical differences in numeric data showing a normal distribution were evaluated using one-way ANOVA followed by Bonferroni post hoc test for comparing study groups. Three high power fields (magnification 100) from five serial sections (fixed 15 µm inter-section) per animal for each group were examined. Data from scoring or quantitative data from PCR results were presented as medians and analyzed by a non-parametric ANOVA and a post hoc test. The differences were considered significant when *p* ≤ 0.05. To approve the statistical power of the experimental parameters after exclusion of dead animals, power analysis was conducted using G*Power computer program (Version 3.1.9.4 © 1992–2019) [66].

## Figures and Tables

**Figure 1:**
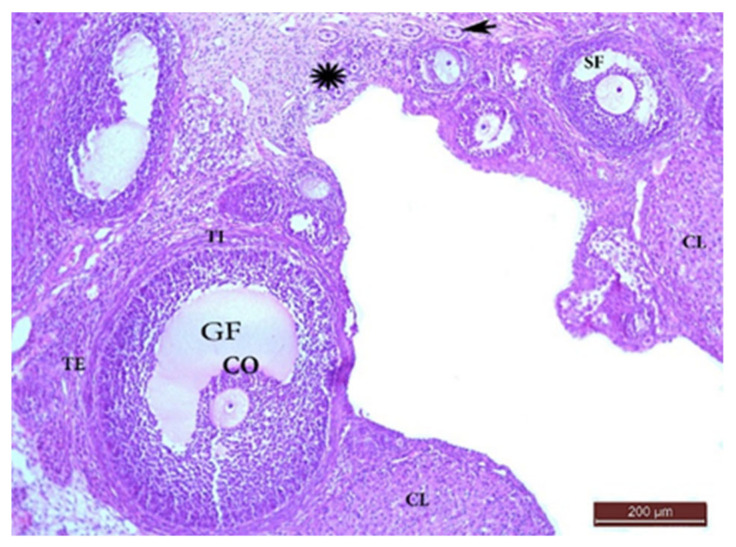
H&E ×100 Photomicrograph from G I ovary. Ovarian section from G I rat at x100 magnification showing different stages of normal follicular development with little stroma in between. Mature Graafian follicle (GF) containing cumulus oophorus (CO) and surrounded with theca interna (TI) and theca externa (TE) layers. Multiple primordial follicles (*), primary follicles (arrow), and secondary follicle (SF) with corpora lutea (CL) had large faintly stained acidophilic cells.

**Figure 2:**
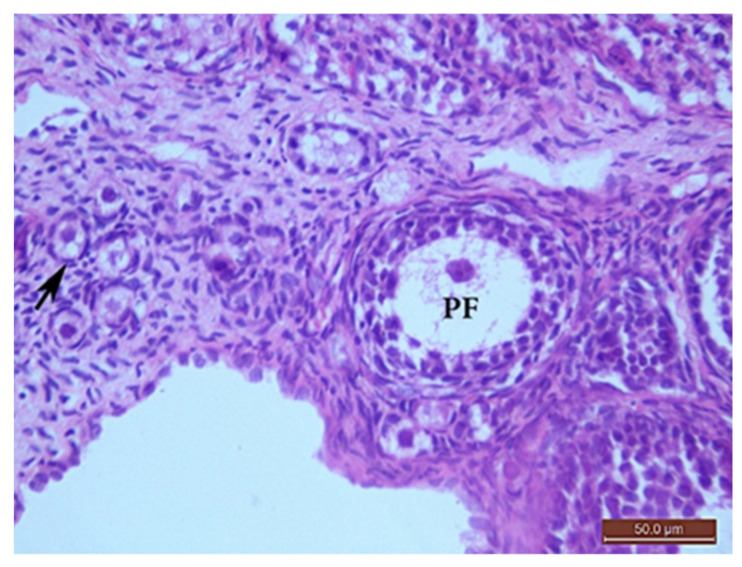
H&E ×400 Photomicrograph from G I ovary. Ovarian section from G I rat at x400 magnification showing a primordial follicle (arrow) with a small, rounded oocyte with obvious nucleus, surrounded by flat stromal cells. The primary follicle (PF) had a bigger oocyte surrounded by one layer of cuboidal cells, one or two layers of granulosa cells and outer flat stromal cells.

**Figure 3:**
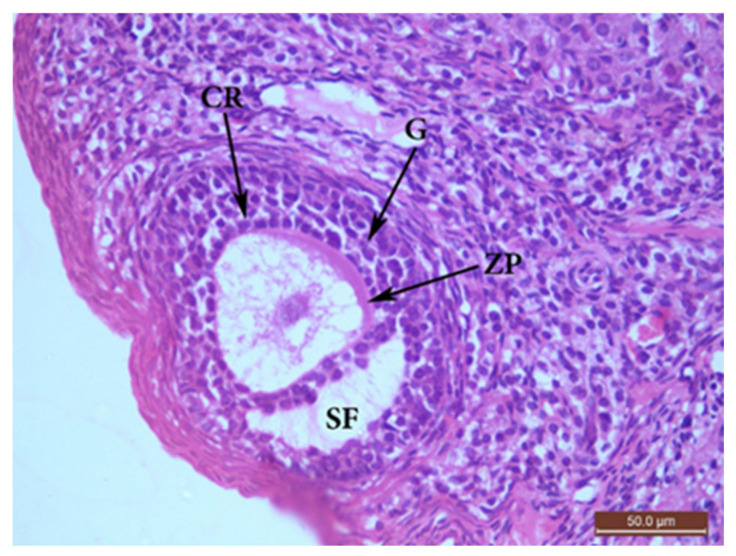
H&E ×400 Photomicrograph from G I ovary. Ovarian section from G I rat at x400 magnification showing a secondary follicle (SF) consisting of a big oocyte with obvious nucleus surrounded by corona radiata (CR) overlying a zona pellucida (ZP), multiple layers of granulosa (G) cells with a fluid cavity. The SF is also surrounded by flat stromal cells.

**Figure 4:**
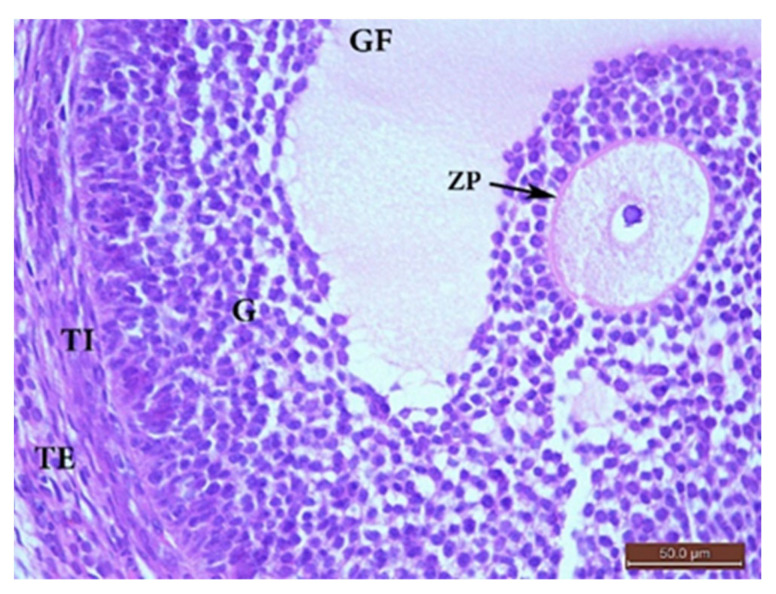
H&E ×400 Photomicrograph from group (I) ovary. Ovaria section from G I rat showing a mature Graafian follicle (GF) surrounded by theca interna (TI) and theca externa (TE), separated from the follicle by a basement membrane. The follicle had a cumulus oophorus (CO) and single big fluid cavity. The CO had a normal oocyte with an obvious nucleus surrounded by a zona pellucida (ZP), corona radiata, and multiple layers of granulosa (G) cells.

**Figure 5:**
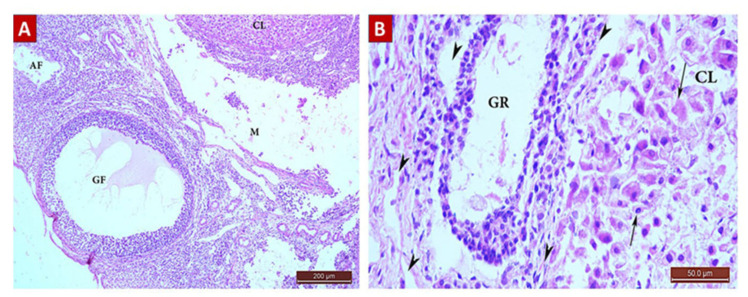
H&E Photomicrograph from The POI (G II) group showing extensive ovarian damage. (**A**) Ovarian section from G II rat at x100 magnification showing a damaged Graafian follicle (GF) with no cumulus oophorus and loss of other follicle stages with multiple atretic follicles (AF). The medulla (M) and cortical tissue showed destruction with edema. (**B**) Ovarian section from G II rat at x400 magnification showing shrinkage of granulosa cells with nuclear pyknosis. The wall of the GF was separated and destructed with edema (arrow heads) with loss of demarcation between its layers. The corpus luteum (CL) shows necrosis, shrinkage, and pyknosis or loss of the cells nuclei (arrows), loss of cells in other areas, and destruction of its wall.

**Figure 6:**
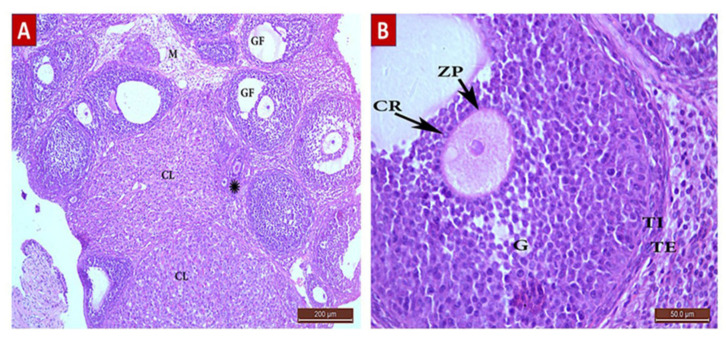
H&E Photomicrograph from G III group showing an improvement in ovarian architecture compared to POI (G II) group. (**A**) Ovarian section from G III group rat at x100 magnification showing little stoma with follicles in different stages of development, primordial and primary follicles (*), multiple Graafian follicles (GF), and corpus lutea (CL) comparable to the control (G I) group. Normal medulla (M) is visible. (**B**) Ovarian section from G III group rat at x400 magnification showing a normal GF with an oocyte surrounded by zona pellucida (ZP), corona radiata (CR), and granulosa (G) cells. The follicle is surrounded by normal layers of theca interna (TI) and theca externa (TE) cells.

**Figure 7:**
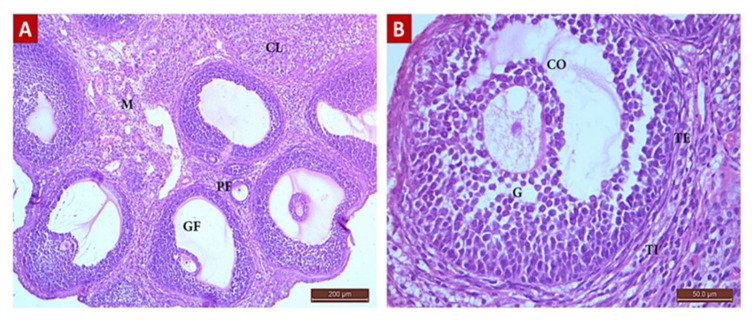
H&E photomicrograph from G IV group showing ovarian architecture comparable to control (G I) group, a remarkable improvement from that shown in POI (G II) group. (**A**) Ovarian section from G IV group rat at x100 magnification showing the ovarian cortex with follicles in different stages of development, primary follicles (PF), multiple Graafian follicles (GF), and corpus luteus (CL) comparable to control (G I) group. Normal medulla (M) is visible. (**B**) Ovarian section from G III group rat at x400 magnification showing a normal GF comprising an antrum, cumulus oophorus (CO), oocyte, and a single fluid cavity lined by granulosa (G) cells. The follicle is surrounded by normal layers of theca interna (TI) and theca externa (TE) cells.

**Table 1:** Body weight and ovarian weight in different groups.

Groups	Body Weight (gm)	Ovarian Weight (gm)
G I	139 ± 15	18 ± 5
G II	130 ± 10	8 ± 2 *
G III	132 ± 22	10 ± 5 *,$
G IV	134 ± 15	15 ± 3 $

Data are expressed as means ± SEM (*n* = 6) and analyzed using one-way ANOVA followed by post-hoc Bonferroni multiple comparison test. (Symbols: *: *p* ≤ 0.05 compared to the control (GI) group; $: *p*≤ 0.05 compared to POI (G II) group). Abbreviations: G I = control group; G II = POI group; G III = cyclophosphamide with diosmin low dose (50 mg/kg) group; G IV = cyclophosphamide with diosmin high dose (100 mg/kg) group.

**Table 2:** Serum hormonal levels of Follicular stimulating hormone (FSH), Estradiol (E2), and Anti- mullerin hormone (AMH) in different groups.

Groups	FSH Level (mIU/mL)	AMH (mIU/mL)	E2 Level (pg/mL)
G I	0.015 ±0.007	0.330 ± 0.148	18.35 ± 1.77
G II	0.052 ± 0.031 *	0.036 ± 0.017 *	8.53 ± 1.02*
G III	0.003 ± 0.003 *,$	0.237 ± 0.124 *,$	12.15 ± 2.51 *,$
G IV	0.011 ± 0.007 $,#	0.284 ± 0.120 *,$	14.68 ± 3.87 *,$

Data are expressed as means ± SEM (*n* = 6) and analyzed using one-way ANOVA followed by post-hoc Bonferroni multiple comparison test. (Symbols: *: *p* ≤ 0.05 compared to the control (G I) group; $: *p* ≤ 0.05 compared to POI (G II) group; #: *p* ≤ 0.05 compared to Diosmin 50 (G IV) group). Abbreviations: G I = control group; G II = POI group; G III = cyclophosphamide with diosmin low dose (50 mg/kg) group; G IV = cyclophosphamide with diosmin high dose (100 mg/kg) group.

**Table 3:** Serum Malonaldehyde (MDA) and catalase activity levels in study groups.

Groups	MDA Level (nmol/mL)	Catalase Activity (U/L)
G I	9.08 ± 0.16 $	78.78 ± 1.98
G II	20.17 ± 7.91 *	59.85 ± 20.08
G III	4.68 ± 1.35 $	83.44 ± 5.37
G IV	6.18 ± 1. 35 $	86.35 ± 2.07

Data are expressed as means ± SEM (*n* = 6) and analyzed using one-way ANOVA followed by post-hoc Bonferroni multiple comparison test. (Symbols: *: *p* ≤ 0.05 compared to the control (G I) group; $: *p* ≤ 0.05 compared to POI (G II) group). Abbreviations: G I = control group; G II = POI group; G III= cyclophosphamide with diosmin low dose (50 mg/kg) group; G IV = cyclophosphamide with diosmin high dose (100 mg/kg) group.

**Table 4:** Mean number of ovarian follicles (per section) in different stages of development.

Groups	Primordial	Primary	Antral	Graafian	Atretic	Total
G I	2.6 ± 1.34	3.8 ± 1.47	2.5 ± 0.52	1.5 ± 0.52	0.6 ± 0.51	11
G II	0.5 ± 0.52 *	0.5 ± 0.52 *	0.4 ± 0.41 *	0.2 ± 0.22 *	2.8 ± 0.63 *	4.4
G III	2 ± 0.86	2.5 ± 0.84	1.5 ± 0.7	0.7 ± 0.48	1 ± 0.8	7.7
G IV	3 ± 1.41	3.2 ± 1.31	1.7 ± 0.81	1.4 ± 0.51 $	0.7 ± 0.67	10

Data are expressed as means ± SEM (*n*= 6) and analyzed using one-way ANOVA followed by post-hoc Bonferroni multiple comparison test. (Symbols: *: *p* ≤ 0.05 compared to the control (G I) group; $: *p* ≤ 0.05 compared to POI (G II) group). Abbreviations: G I = control group; G II = POI group; G III= cyclophosphamide with diosmin low dose (50 mg/kg) group; G IV = cyclophosphamide with diosmin high dose (100 mg/kg) group.

## Data Availability

The data that support the findings of this study are available from the corresponding author upon reasonable request.
